# Comprehensive serum, follicular fluid, and ovarian tissue profiling reveals systemic metabolic alterations in high-yielding dairy cows with postpartum inactive ovaries

**DOI:** 10.1186/s12917-025-05153-5

**Published:** 2026-01-05

**Authors:** Yuxi Song, Xuejie Jiang, Yu Hao, Rui Sun, Yunlong Bai, Chuang Xu, Cheng Xia

**Affiliations:** 1 Heilongjiang Provincial Key Laboratory of Prevention and Control of Bovine Diseases, College of Animal Science and Veterinary Medicine, Heilongjiang Bayi Agricultural University, Daqing, 163319 China; 2 College of Veterinary Medicine, China Agricultural University, Yuan Ming Yuan West Road No. 2, Haidian District, Beijing, 100193 China

**Keywords:** Dairy cow, Inactive ovary, Metabolomics, LC–MS/MS, Serum, Follicular fluid, Ovary

## Abstract

**Background:**

Inactive ovaries (IO) commonly cause postpartum anestrus in high-yielding dairy cows. Despite the widespread reporting of single-metabolic characteristics of postpartum IO, a comprehensive metabolic profile is lacking. Liquid chromatography–tandem mass spectrometry was used to compare the metabolic changes in the serum, follicular fluid (FF), and ovarian tissue of six multiparous Holstein cows from each of the IO and healthy control (HC) groups.

**Results:**

Cows with IO had a higher milk yield during the previous lactation, poorer BCS, smaller largest follicle (LF) diameter at 63 ± 3 days in milk, and slower LF growth rate than HC cows (*p* < 0.01). They also exhibited higher serum levels of non-esterified fatty acids and aspartate aminotransferase and lower levels of estradiol, progesterone, insulin-like growth factor 1, calcium, and phosphorus (*p* < 0.01). Under IO conditions, 40, 51, and 14 differential metabolites were identified in serum, FF, and ovarian tissues, respectively. 12-Methyltridecanoic acid was consistently upregulated in cows with IO compared to HC cows across all samples (*p* < 0.01). Metabolomic pathway analysis identified significant alterations in six, three, and two metabolic pathways related to IO in the serum, FF, and ovarian tissues, respectively, affecting amino acid, energy, carbohydrate, lipid, and nucleotide metabolism. Valine, leucine, and isoleucine biosynthesis showed significant changes (*p* < 0.05) in all samples.

**Conclusion:**

In summary, these metabolic changes in cows with IO reflect a complex response to metabolic, oxidative, and inflammatory stresses. Our study provides the most comprehensive metabolic profile for cows with postpartum IO.

## Introduction

Inactive ovaries (IO) is a prevalent postpartum ovarian condition in high-yielding dairy cows, with an 8.5% incidence rate during lactation in the absence of intervention [1]. Around 20% of dairy cows in anestrus exhibit IO at the beginning of breeding program [2] or 63 days in milk (DIM) [3]. The IO is characterized by transient ovarian dysfunction and loss of periodic ovarian follicular activity [4]. This condition, in turn, can extend calving to the first service interval, conception interval (days open), and calving interval [5–7]. Affected cows may subsequently face a higher risk of culling owing to poor reproductive performance, resulting in huge losses for the dairy industry [7].

Historically, IO has been referred to using various terms, including inactive-static ovaries [8], ovarian inactivity [9, 10], ovarian hypofunction [11, 12], and ovarian quiescence [13, 14]. Currently, the widely accepted definition of IO is that both ovaries have no follicles larger than 8–10 mm, no corpus luteum, and the condition lasts longer than seven days [6, 7, 11, 15, 16]. Moreover, cows with IO have serum progesterone concentrations less than 1 ng/mL [5, 15]. Many factors can contribute to IO in cows, including poor body condition score (BCS), inadequate feed intake, excessive milk yield, and improper management of stress and disease [5, 17]. A negative energy balance (NEB), associated with metabolic and hormonal alterations during early lactation, is increasingly recognized as an important contributor to IO [18]. Our recent study highlighted the contributing role of NEB in the development of IO [7]. Thus, preventing and treating IO have become crucial challenges for early lactation management in modern dairy farms.

Follicular fluid (FF) contains bioactive molecules that play an important role in follicular growth and oocyte maturation [19]. The composition of FF changes as the follicle develops, reflecting its pivotal role in oocyte meiosis, ovulation, and fertilization [20]. Similarly, the ovaries provide a localized environment conducive to follicular growth and hormone production [21]. Granulosa cells in the ovaries are vital for the folliculogenesis and steroidogenesis [22]. Metabolites in the FF derived from blood and cellular secretions, as well as those in ovarian tissue, necessitate comparative metabolic analyses of FF, serum, and ovarian tissue in cows with IO to understand the reproductive disorder processes underlying cattle infertility [23].

Recent advances in metabolomics have opened up new avenues for understanding the complex biochemical networks involved in these processes. Metabolomics, the in-depth study of metabolites in biological systems, is a powerful tool for elucidating metabolic alterations associated with specific physiological states or disorders [24]. The pathophysiology of IO in dairy cows is multifaceted and involves complex interactions between the metabolic, endocrine, and immune pathways [25]. Energy metabolism, liver function, and reproductive hormone regulation are critical factors that influence ovarian activity [26]. Previous studies have demonstrated that cows with IO exhibit distinct serum, whey, and FF metabolic profiles compared to their estrus counterparts, highlighting the capability of metabolomics in discovering biomarkers for early diagnosis and intervention [9, 27].

Despite the importance of reproductive health in dairy cows, limited information exists regarding the metabolomic changes associated with IO. Previous studies have primarily focused on hormonal and biochemical parameters [7, 28, 29], as well as metabolic profiles based on one or two types of samples [4, 9, 27]; however, comprehensive metabolomic analysis is lacking. Understanding the metabolic basis of IO may contribute to creating diagnostic instruments and treatment approaches aimed at enhancing the reproductive efficiency of dairy cows. Identifying specific metabolic markers associated with IO may facilitate early diagnosis and targeted treatment, thereby enhancing reproductive efficiency and reducing economic losses [30]. This study aimed to comprehensively analyze serum, FF, and ovarian tissue samples using liquid chromatography-tandem mass spectrometry (LC–MS/MS) to investigate the metabolic differences between postpartum Holstein dairy cows experiencing IO and those that are healthy. Hypothetically, specific biomarkers of IO exist in serum, and changes in their levels reflect those in FF or ovarian tissue. Such biomarkers could be used to develop novel and reliable diagnostic tools for IO, enhancing early intervention and effective management for affected cows. This study provides the most thorough metabolomic evaluation of IO in cows, offering a broader context for its supposition.

## Materials and methods

### Animal handling and management

This study was carried out with Holstein cows from a substantial intensive cattle farm in central Heilongjiang Province, China, following the guidelines of the Veterinary Medical Ethics Committee of the Ministry of Agriculture of China. Approval was obtained from the Animal Welfare and Research Ethics Committee of Heilongjiang Bayi Agricultural University (protocol code: DWKJXY2024007; approval date: January 4, 2024). The farm has a population of 3,215 cows, including 1,671 lactating animals. The average parity of the cows was 3.05 ± 1.12, with an average annual milk yield of 12,010 ± 1,548 kg (milk fat, 4.61 ± 0.32%; milk protein, 3.33 ± 0.22%; mean *±* standard deviation [SD]). During the experimental period, 701 cows calved, and the incidence rate of postpartum IO was approximately 5.7% after estrus synchronization. From these, potential candidates were screened based on parity (multiparous), absence of major health issues, and completion of estrus synchronization. Initially, 16 cows met the preliminary criteria; however, after thorough veterinary examination, four cows were excluded due to other health problems (two with ketosis, one with left displaced abomasum, and one with mastitis), resulting in 12 cows that were finally enrolled in the study. These 12 multiparous Holstein cows (age, 3.98 ± 0.62 years; parity, 2.75 ± 0.62; BCS, 2.88 ± 0.39; daily milk yield during the first 63 DIM, 45.54 ± 2.18 kg/d; milk yield during the previous lactation, 13,130.00 ± 1,185.63 kg; mean *±* SD) were divided into a healthy control (HC) group (*n* = 6) and an IO group (*n* = 6) based on two consecutive rectal palpation and ultrasonography examinations at 56 ± 3 and 63 ± 3 DIM. Corresponding data (cow identification number, herd code, and calving date) were recorded. The cows were raised in the same environment, fed the same diet, unrestricted access to fresh water, and uniform same management program. During the 60-day dry period, the cows were fed a total mixed ration (TMR) diet twice daily. After calving, the cows were milked three times each day (0500, 1300, and 2200 h) and fed a TMR diet three times each day (0600, 1400, and 2300 h) primarily consisting of corn silage. National Research Council (NRC, 2001) guidelines were followed in the formulation of the TMR for early lactation [31]. Table 1 presents the components and chemical composition of the TMR diet during early lactation. Specific software (Afifarm version 5.3; Afimilk, Kibbutz Afikim, Israel) was utilized to document the age, parity, and milk yield of the dairy cows. A five-point scoring method using 0.25-unit intervals was used to evaluate the BCS of the dairy cows by two well-trained veterinarians [32].

**Table 1:** Ingredients and chemical composition of the diets for early lactating dairy cows

Item	Early lactation period
Ingredient, % of dry matter	
Water	11.18
Cottonseed	2.10
Soybean husk	3.06
Oat grass	1.02
Alfalfa	5.11
Silage	58.54
Corn	7.76
Soybean meal	2.66
Flaked corn	4.08
Molasses	2.04
Fat powder	0.82
Premix^1^	1.63
Nutrient, % of dry matter	
Dry matter	55.60
Crude protein	16.00
Acid detergent fiber	20.30
Neutral detergent fiber	39.10
Calcium	0.40
Phosphorus	0.30

^1^ Concentration per kilogram of premix DM: 40,000 IU of vitamin A, 37,000 IU of vitamin D, 500 IU of vitamin E, 30 mg of copper, 25 mg of iron, 140 mg of manganese, 140 mg of zinc, 0.8 mg of selenium

### Estrous synchronization protocol

The first artificial insemination was performed using the Presynch-Ovsynch protocol on all cows [33]. In brief, the procedure began with a 25 mg intramuscular injection of prostaglandin F2 alpha (PGF_2α_) (Lutalyse; dinoprost tromethamine; Pfizer Animal Health, New York, NY, USA) on 30 ± 3 DIM, and was subsequently followed by a second 25 mg injection of PGF_2α_ after a period of 14 days (on average of 44 ± 3 DIM). Twelve days post the second PGF_2α_ administration (on average of 56 ± 3 DIM), the ovulation synchronization protocol commenced with the administration of 100 µg of gonadotropin-releasing hormone (GnRH) (Cystorelin; gonadorelin diacetate tetrahydrate; Merial, Duluth, GA, USA), followed by a third PGF_2α_ (25 mg) injection seven days later (on average of 63 ± 3 DIM). A final dose of GnRH (100 µg) was given 56 h after the third PGF_2α_ injection (on average of 65 ± 3 DIM), but no insemination occurred 16 to 18 h later (on average of 66 ± 3 DIM).

### Ovarian ultrasonography

At 56 ± 3 and 63 ± 3 DIM, all cows were examined for ovarian structures and activity using transrectal ultrasonography (Easi-Scan Curve, 5.0 MHz; BCF Technology Ltd., Bellshill, UK), as described previously [7]. Briefly, the presence of a typical corpus luteum and/or sizable follicles (>10 mm) in two ultrasonographic assessments of the ovaries conducted seven days apart, along with a serum progesterone level above 1 ng/mL, were included in the HC group. Cows in the HC group were all in the luteal phase. The IO group included cows that did not have ovarian cysts, persistent corpus luteum, or follicles, as well as those lacking a normal corpus luteum and/or large follicles (>10 mm). Two ultrasonographic assessments of the ovaries, conducted seven days apart, indicated that there were no significant changes in the follicular structures, and the serum progesterone concentration was < 1 ng/mL. The diameter of the largest follicle (LF) in the ovaries of each cow was measured during both ultrasonographic examinations (at 56 ± 3 and 63 ± 3 DIM). It should be noted that these LFs were derived from new follicular waves induced by the first GnRH administration, as the protocol effectively synchronizes follicular development. The final daily growth rate of the follicles was computed by taking the difference between the LF diameters at 63 ± 3 and 56 ± 3 DIM and dividing this value by seven.

### Sample collection

At 63 ± 3 DIM, blood samples were obtained from cows in both the IO and HC groups through the tail vein using disposable vacuum blood collection tubes (5 mL; Kangweishi Medical Technology Co., Ltd, Hebei, China) without anticoagulants. Following a 30-min clotting period at room temperature, a centrifuge was used to centrifuge samples for 10 min at 3,500 × g. The serum obtained was subsequently maintained at −80℃ for later biochemical and LC–MS/MS analyses. Ovarian FF and ovarian cortical tissue samples were independently harvested from the same ovary in each cow using transvaginal ultrasound-guided puncture and aspiration puncture (Easi-Scan Curve, 5.0 MHz), as previously described [27, 34]. Briefly, a FF sample (100–200 µL) was aspirated using a puncture needle from the LF in the ovaries of each cow under ultrasound guidance. An ovarian cortical tissue sample (50–100 mg) was subsequently harvested from the specimen notch of the puncture needle. A centrifuge was used to centrifuge FF samples for 10 min at 12,000 × g, and the supernatant was maintained at −80℃ for future LC–MS/MS analysis. The tissues were grouped, labeled, packed into sterile freezing tubes, quickly frozen in liquid nitrogen, and maintained at −80℃ until analysis by LC–MS/MS.

### Serum biochemical detection

Serum concentrations of β-hydroxybutyrate, non-esterified fatty acids (NEFA), glucose, aspartate aminotransferase (AST), alanine aminotransferase (ALT), total protein (TP), calcium, phosphorus, and magnesium were measured using commercial biochemical assay kits provided by Mindray Biomedical Electronics Co., Ltd. (Shenzhen, China) and analyzed with the Mindray BS-830 S fully automatic biochemistry analyzer (Mindray Biomedical Electronics Co., Ltd.) at the Biotechnology Centre of Heilongjiang Bayi Agricultural University. All analyses were performed according to the instructions of manufacturer.

Serum concentrations of estradiol and progesterone were assessed utilizing two commercial kits from the same batch (Xinfan Biotechnology Co., Ltd., Shanghai, China) and quantified using an XH-6080 radioimmunocounter (Xian Nuclear Instrument Co., Ltd., Xian, China) following the validated radioimmunoassay protocols established by the manufacturer [35]. There was a sensitivity of 2 pg/mL for estradiol and 0.2 ng/mL for progesterone in the assays. The intra-assay coefficient of variation was < 10% and the inter-assay coefficient of variation was < 15%.

Serum insulin-like growth factor 1 (IGF-1) levels were determined using a bovine-specific ELISA kit (Xinfan Biotechnology Co., Ltd.). Quantification was carried out with a Multiskan FC microplate reader (Thermo Fisher Scientific Co., Ltd., Shanghai, China) at a wavelength of 450 nm, following the manufacturer’s guidelines.

### Sample preparation

Preparation of samples for LC-MS/MS analysis was carried out according to the previous description [27, 36]. In brief, 100 µL of serum or FF was combined with 300 µL of methanol (LC-MS grade) including 1 µg/mL L-2-chlorophenylalanine, serving as the internal standard. The sample was subjected to vortex mixing for 30 s, followed by sonication for 10 min in an ice-water bath. It was then incubated at −40℃ for 1 h to allow protein precipitation, after which it was centrifuged at 12,000 × g for 15 min at 4℃. The supernatant was carefully transferred to a new glass vial for subsequent LC-MS/MS analysis. Ovarian cortical tissue was ground in liquid nitrogen. Fifty milligrams of the ground sample was combined with 1,000 µL of extraction solvent (methanol (LC-MS grade): acetonitrile (LC-MS grade): water (LC-MS grade) in a 2:2:1 ratio) including 1 µg/mL L-2-chlorophenylalanine as the internal standard, vortexed for 30 s, homogenized at 35 Hz for 4 min, and sonicated for 5 min on ice. The cycles of homogenization and sonication were carried out three times. The mixture was then incubated for 1 h at −40℃, followed by centrifugation at 12,000 × g at 4℃ for 15 min, and the supernatant obtained was collected into a new sampling bottle for LC–MS/MS analysis. To prepare a quality control sample, equal volumes of supernatants from all samples were combined.

### LC–MS/MS analysis

The LC–MS/MS analyses were conducted utilizing a Vanquish UHPLC system (Thermo Fisher Scientific) equipped with a UPLC BEH Amide column (2.1 mm × 100 mm, 1.7 μm) connected to a Q Exactive HFX mass spectrometer (Orbitrap MS, Thermo Fisher Scientific). The mobile phase comprised 25 mmol/L ammonium acetate and 25 mmol/L ammonia hydroxide in water (pH 9.75) (A) and acetonitrile (B). The elution gradient was as follows: from 0 to 0.5 min, 95% B; from 0.5 to 7.0 min, a linear decrease from 95% to 65% B; from 7.0 to 8.0 min, a further decrease from 65% to 40% B; from 8.0 to 9.0 min, maintained at 40% B; from 9.0 to 9.1 min, a rapid increase from 40% to 95% B; and from 9.1 to 12.0 min, maintained at 95% B. The mobile phase flowed at a rate of 0.5 mL/min, with the column maintained at 35 °C and the autosampler at 4 °C, while the injection volume was set to 3 µL. A QE HFX mass spectrometer was utilized due to its capability to obtain MS/MS spectra in an information-dependent acquisition mode, managed by the acquisition software (Xcalibur, Thermo Fisher Scientific). In this mode, the software for acquisition consistently assessed the complete mass spectrometry spectrum obtained from full scans. ESI source parameters were configured as follows: sheath gas flow rate, 50 Arb; Aux gas flow rate, 10 Arb; capillary temperature at 320℃. The full MS resolution was established at 60,000, while the MS/MS resolution was set to 7,500. Collision energy values were 10/30/60 in NCE mode, and the spray voltage was configured to 3.5 kV for positive-ion mode or −3.2 kV for negative-ion mode.

### Data processing and multivariate statistical analyses

ProteoWizard was used to convert raw data to mzXML [37]. R package XCMS was used to align peak areas, adjust retention times, and extract peak areas [38]. For the data processed with XCMS, ion peak data with more than 50% missing values within a group were deleted. After Pareto-scaling preprocessing, pattern recognition was carried out utilizing SIMCA-P software (version 14.1; Umetrics, Umea, Sweden). This analysis encompassed unsupervised principal component analysis (PCA) as well as supervised orthogonal partial least squares discriminant analysis (OPLS-DA). PCA was employed to assess the aggregation of data within groups and the separation between groups, while OPLS-DA was utilized to investigate inter-group differences in greater detail. Validation of the OPLS-DA models was achieved using variations in Y (R^2^Y) and the model forecasting ability (Q^2^) using cross-validation and permutation tests with 200 iterations. Models were considered stable and reliable when 1 ≥ R^2^Y and Q^2^ ≥ 0.4 [39]. Additionally, a Q^2^ intercept of less than 0.05 in the permutation test was applied to check for overfitting [40]. Univariate analyses were additionally conducted, incorporating Student’s t-test and fold-change (FC) analysis.

### Metabolite identification and pathway analysis

Metabolites with significantly different levels between groups were identified based on variable importance in the projection (VIP) scores (VIP ≥ 1.5) derived from the OPLS-DA model, along with FC criteria (FC ≥ 2 or FC ≤ 0.5) from univariate analysis, and *p*-values (*p* < 0.05) obtained from the Student’s t-test. Differential metabolites were visualized utilizing bar graphs and Venn diagrams created with GraphPad Prism 9.0 (GraphPad Software Inc., San Diego, CA, USA). An Euclidean distance matrix was established for the mathematical analysis of differential metabolites in each series of analyses. Divergent metabolites were clustered using the complete linkage method, which was illustrated as a heatmap representing hierarchical clustering. The Human Metabolome Database (https://hmdb.ca/, last accessed on September 30, 2024) and Kyoto Encyclopedia of Genes and Genomes (KEGG) database (www.kegg.jp/kegg/pathway.html, last accessed on September 30, 2024) were used to identify the differential metabolites associated with various metabolic pathways. The results of the metabolic pathway analysis were presented in a bubble plot. The clustering heatmap and bubble plot for the differential metabolites were visualized using MetaboAnalyst 6.0 (https://new.metaboanalyst.ca/; last accessed on September 30, 2024).

### Statistical analysis

Each individual cow was considered an experimental unit. Statistical analyses were conducted utilizing IBM SPSS Statistics for Windows (version 26.0; IBM Corp., Armonk, NY, USA). An independent-samples Student’s t-test was utilized to assess differences in clinical and serum biochemical variables between the IO and HC groups. “NormalizeData” function was used to normalize the non-metabolomics (clinical/biochemical) data. No data points exceeded three SDs from the mean for any measured variable, thus all six animals per group were retained for analysis. All non-metabolomics data were confirmed to be normally distributed using Shapiro-Wilk tests (*p* > 0.05 for all variables), justifying our use of parametric tests. Data are expressed as mean ± SD. Statistical significance was defined as *p* < 0.05.

## Results

### Clinical characteristics and serum biochemical measurements

The clinical characteristics and serum biochemical indicators of the IO and HC groups are presented in Table 2. Although both groups were similar in age, parity, milk yield during the first 63 DIM, and LF diameter at 56 ± 3 DIM (*p* > 0.05), cows in the IO group exhibited a higher milk yield during the previous lactation, along with a lower BCS, a smaller LF diameter at 63 ± 3 DIM, and a slower LF growth rate (*p* < 0.01). Additionally, the IO group exhibited higher serum NEFA and AST levels and lower serum estradiol, progesterone (0.53 ± 0.15 vs. 6.37 ± 2.18 ng/mL), IGF-1, calcium, and phosphorus levels than the HC group (*p* < 0.01). No significant differences were observed in the other serum biochemical parameters between the groups (*p* > 0.05).

**Table 2:** Clinical characteristics and serum biochemical indicators of the IO and HC groups

Item	IO group (*n* = 6)	HC group (*n* = 6)	*p*-value
Age (years)	4.13 ± 0.78	3.83 ± 0.41	0.426
Parity	2.83 ± 0.75	2.67 ± 0.52	0.664
BCS	2.58 ± 0.13	3.17 ± 0.34	0.007
Milk yield during the first 63 DIM (kg/d)	44.73 ± 1.31	46.35 ± 2.67	0.211
Milk yield during the previous lactation (kg)	14,133.33 ± 744.76	12,126.67 ± 349.09	< 0.001
LF diameter at 56 ± 3 DIM (mm)	3.50 ± 0.84	4.00 ± 0.89	0.341
LF diameter at 63 ± 3 DIM (mm)	7.50 ± 1.22	12.83 ± 1.60	< 0.001
LF growth rate (mm/d)	0.57 ± 0.25	1.26 ± 0.10	< 0.001
Estradiol concentration (pg/mL)	7.29 ± 1.08	20.78 ± 3.82	< 0.001
Progesterone concentration (ng/mL)	0.53 ± 0.15	6.37 ± 2.18	0.001
IGF-1 concentration (ng/mL)	27.63 ± 2.47	84.22 ± 8.94	< 0.001
BHB concentration (mmol/L)	0.80 ± 0.39	0.75 ± 0.23	0.793
NEFA concentration (mmol/L)	0.55 ± 0.02	0.39 ± 0.05	< 0.001
Glucose concentration (mmol/L)	3.19 ± 0.02	3.28 ± 0.23	0.409
AST activity (U/L)	81.67 ± 10.46	56.17 ± 10.30	0.002
ALT activity (U/L)	20.00 ± 4.15	24.67 ± 10.38	0.331
TP concentration (g/L)	63.63 ± 14.16	59.32 ± 14.38	0.612
Calcium concentration (mmol/L)	2.04 ± 0.03	2.24 ± 0.07	< 0.001
Phosphorus concentration (mmol/L)	1.55 ± 0.13	2.01 ± 0.25	0.003
Magnesium concentration (mmol/L)	1.14 ± 0.14	1.12 ± 0.07	0.815

*IO* inactive ovaries, *HC* healthy control, *BCS* body condition score, *DIM* days in milk, *LF* largest follicle, *IGF-1* insulin-like growth factor 1, *BHB* β-hydroxybutyric acid, *NEFA* non-esterified fatty acids, *AST* aspartate aminotransferase, *ALT* alanine aminotransferase, *TP* total protein

### Multivariate statistical analysis

**Fig. 1:**
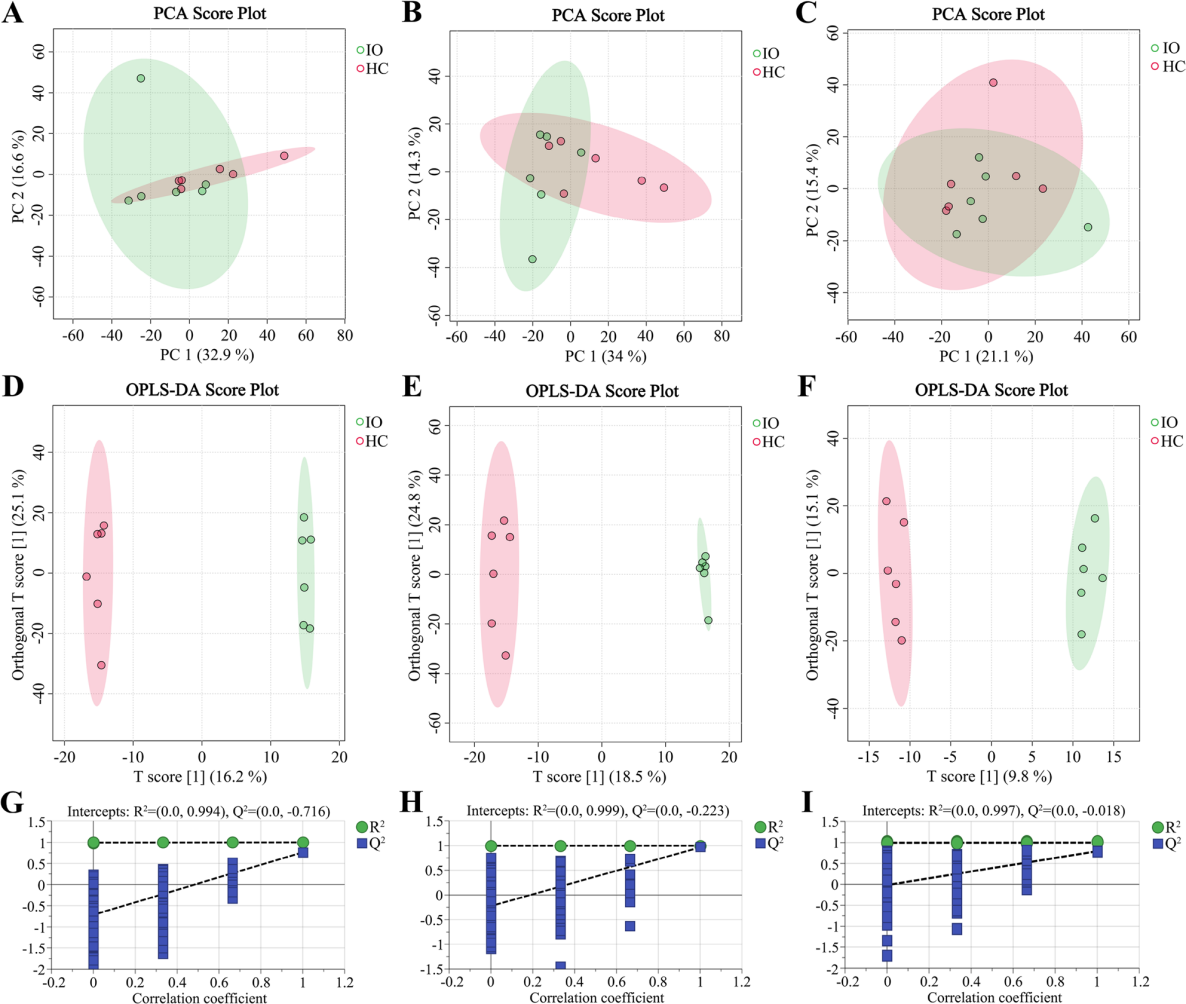
Multivariate statistical analyses of metabolites between the IO group (*n* = 6) and the HC group (*n* = 6). The PCA score plots for the serum (**A**), FF (**B**), and ovarian tissue (**C**) samples, respectively. The OPLS-DA score plots for the serum (**D**), FF (**E**), and ovarian tissue (**F**) samples, respectively. Permutation test plots (200 permutation tests) for the OPLS-DA model of the serum (**G**), FF (**H**), and ovarian tissue (**I**) samples, respectively. IO, inactive ovaries; HC, healthy control; PCA, principal component analysis; FF, follicular fluid; OPLS-DA, orthogonal partial least squares discriminant analysis; R^2^Y, goodness-of-fit parameter; Q^2^, predictive ability parameter

A total of 12,570, 11,352, and 12,643 peaks were obtained in the serum, FF, and ovarian tissue samples, respectively. Subsequent screening of these peaks led to the identification of 1,542 metabolites in the serum, 1,536 metabolites in the FF, and 1,569 metabolites in the ovarian tissue. Unsupervised PCA was utilized to observe the overall distribution of samples (Fig. 1A, B, and C). The PCA score plots indicated a partial overlap of the serum, FF, and ovarian tissue samples between the IO and HC groups. To better distinguish between the IO and HC groups and enhance the effectiveness and analytical capability of the model, supervised OPLS-DA analysis was conducted (Fig. 1D, E, and F). According to the OPLS-DA score plots, the IO and HC groups were significantly different, indicating notable differences in metabolites between the groups. The R^2^Y and Q^2^ values of the OPLS-DA score plots for the serum, FF, and ovarian tissue samples were 1 and 0.965, 1 and 0.966, and 1 and 0.792, respectively, all of which were close to 1, suggesting that the model was both stable and reliable. To assess the risk of overfitting in the OPLS-DA model, its quality was examined using the 200-response permutation test. The Q^2^ intercept values were less than 0.05, indicating there no overfitting (Fig. 1G, H, and I).

### Differential metabolite analysis

**Fig. 2:**
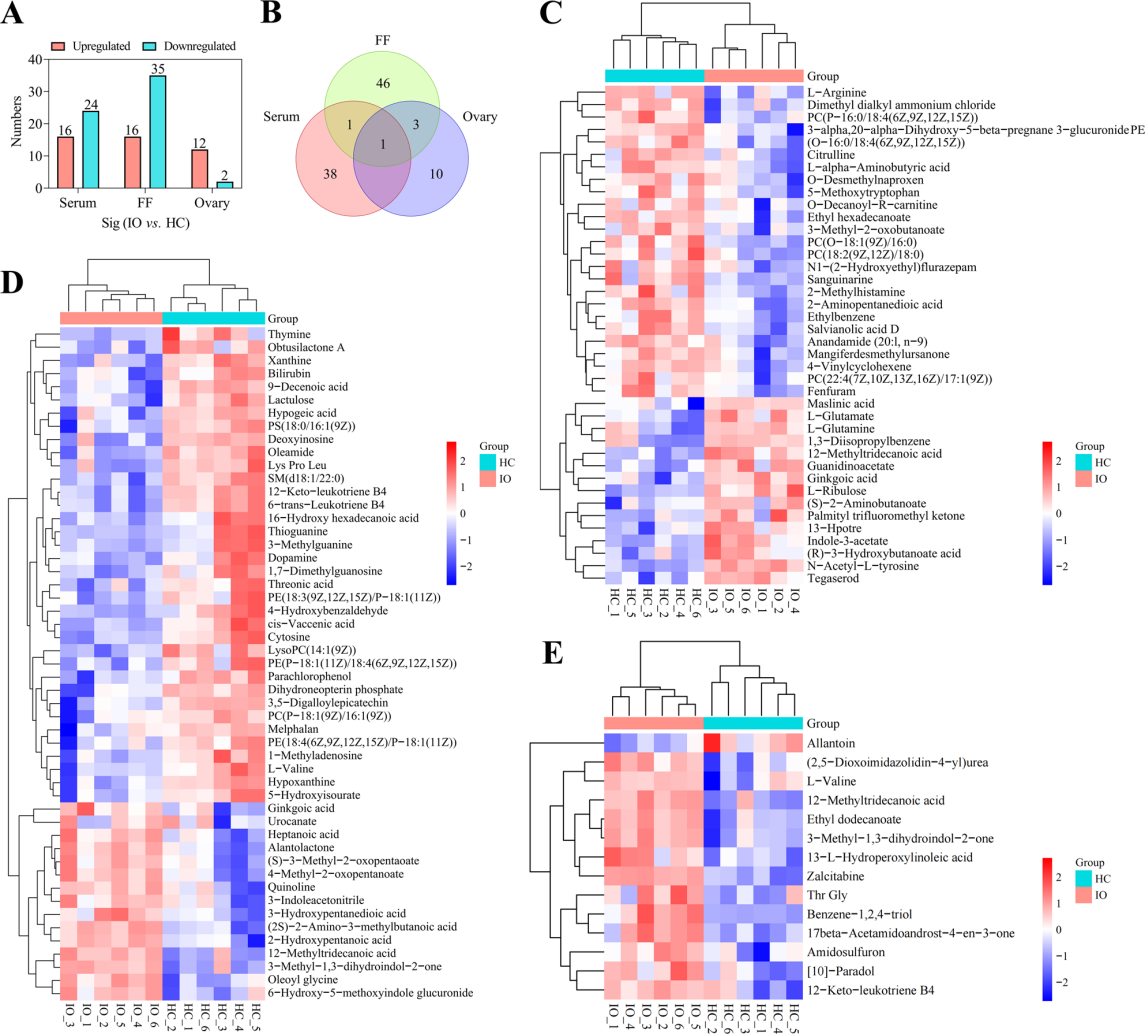
Identification and comparison of differential metabolites between the IO group (*n* = 6) and the HC group (*n* = 6). **A** Statistical bar charts of differential metabolites for the serum, FF, and ovarian tissue samples, respectively. **B** Venn analysis of differential metabolites among the serum, FF, and ovarian tissue samples. Clustering heatmap of the 40, 51, and 14 differential metabolites identified in the serum (**C**), FF (**D**), and ovarian tissue (**E**) samples, respectively. IO, inactive ovaries; HC, healthy control; FF, follicular fluid

Differential metabolite screening was conducted using the criteria of VIP ≥ 1.5, FC ≥ 2 or FC ≤ 0.5, and *p* < 0.05. Table 3 shows the upregulation and downregulation of differential metabolites in the serum, FF, and ovarian tissues of cows in the IO group relative to the HC group. Forty differential metabolites were identified in the serum samples, of which 16 were upregulated and 24 were downregulated (Fig. 2A). Fifty-one differential metabolites were identified in the FF samples, of which 16 were upregulated and 35 were downregulated (Fig. 2A). Fourteen differential metabolites were identified in the ovarian tissue, with 12 upregulated and two downregulated metabolites (Fig. 2A). Moreover, the differential metabolites identified in both the serum and FF samples included ginkgoic acid and 12-methyltridecanoic acid (Fig. 2B; Table 3). Only 12-methyltridecanoic acid was detected at differential levels in both serum and ovarian tissues (Fig. 2B; Table 3). The ovarian tissue and FF contained differential levels of L-valine, 3-methyl-1,3-dihydroindol-2-one, 12-keto-leukotriene B4 (12-Keto-LTB4), and 12-methyltridecanoic acid (Fig. 2B; Table 3). Notably, 12-methyltridecanoic acid was upregulated in the IO group compared to the HC group in serum, FF, and ovarian tissue samples (*p* < 0.01, Fig. 2B; Table 3). The relative abundances of 40, 51, and 14 differential metabolites in the serum, FF, and ovarian tissue were compared between the IO and HC groups, respectively, revealing a distinct clustering pattern on a heatmap (Fig. 2C, D, and E).

**Table 3:** Differential metabolites in the serum, FF, and ovarian tissue of the IO and HC groups

Category	Metabolites names	m/z	RT (min)	FC	VIP	*p*-value	IO vs. HC	Mode
Serum	12-Methyltridecanoic acid	227.021	27.006	2.882	2.480	0.0051	up	ESI−
	Ethyl hexadecanoate	283.155	115.383	0.369	1.957	0.0013	down	ESI−
	Indole-3-acetate	173.081	149.562	5.658	2.473	0.0405	up	ESI−
	Palmityl trifluoromethyl ketone	307.055	90.461	2.052	1.852	0.0498	up	ESI−
	Ginkgoic acid	345.244	54.908	2.157	2.150	0.0045	up	ESI−
	(R)−3-Hydroxybutanoate acid	105.100	206.156	2.722	2.100	0.0269	up	ESI−
	3-alpha,20-alpha-Dihydroxy-5-beta-pregnane 3-Glucuronide PE	495.298	235.048	0.494	2.002	0.0038	down	ESI−
	N-Acetyl-L-tyrosine	222.077	213.200	2.255	2.725	< 0.0001	up	ESI−
	(s)−2-Aminobutanoate	104.120	216.705	2.547	1.688	0.0131	up	ESI−
	O-Desmethylnaproxen	214.076	246.812	0.464	1.822	0.0333	down	ESI−
	L-Ribulose	149.045	315.920	2.090	2.523	0.0086	up	ESI−
	Tegaserod	299.186	296.379	2.974	2.162	0.0031	up	ESI−
	13-Hpotre	308.621	55.831	2.532	2.083	0.0131	up	ESI−
	3-Methyl-2-oxobutanate	115.039	64.893	0.458	1.829	0.0300	down	ESI−
	Maslinic acid	472.086	26.180	3.704	1.677	0.0006	up	ESI−
	N1-(2-Hydroxyethyl)flurazepam	333.079	310.260	0.457	1.627	0.0250	down	ESI+
	L-Glutamine	147.151	54.520	3.110	1.553	0.0227	up	ESI+
	Citrulline	177.025	429.128	0.470	1.628	0.0060	down	ESI+
	L-Glutamate	148.131	58.82	2.233	1.529	0.0101	up	ESI+
	1,3-Diisopropylbenzene	163.123	5.006	2.882	1.545	0.0261	up	ESI+
	L-alpha-Aminobutyric acid	104.071	345.354	0.458	1.516	0.0151	down	ESI+
	Dimethyl dialkyl ammonium chloride	303.309	54.534	0.374	1.517	0.0041	down	ESI+
	O-decanoyl-R-carnitine	316.161	242.305	0.492	1.522	0.0491	down	ESI+
	2-Aminopentanedioic acid	147.612	393.743	0.412	1.810	0.0017	down	ESI+
	Mangiferdesmethylursanone	429.373	45.972	0.319	1.615	0.0351	down	ESI+
	Ethylbenzene	107.049	24.836	0.449	1.589	0.0174	down	ESI+
	PC(22:4(7Z,10Z,13Z,16Z)/17:1(9Z))	821.627	149.422	0.493	1.551	0.0449	down	ESI+
	Guanidoinoacetate	118.121	418.864	2.021	1.361	0.0461	up	ESI+
	Anandamide (20:l, n-9)	354.279	111.179	0.450	1.678	0.0062	down	ESI+
	5-Methoxytryptophan	235.108	69.179	0.456	1.537	0.0201	down	ESI+
	PE(O-16:0/18:4(6Z,9Z,12Z,15Z))	698.513	160.647	0.460	1.629	0.0051	down	ESI+
	PC(P-16:0/18:4(6Z,9Z,12Z,15Z))	737.580	166.753	0.373	1.777	0.0270	down	ESI+
	PC(O-18:1(9Z)/16:0)	746.603	158.096	0.347	1.898	0.0190	down	ESI+
	2-Methylhistamine	126.103	28.055	0.396	1.662	0.0432	down	ESI+
	4-Vinylcyclohexene	108.393	51.885	0.430	1.714	0.0041	down	ESI+
	PC(18:2(9Z,12Z)/18:0)	786.658	198.720	0.483	1.529	0.0455	down	ESI+
	L-Arginine	175.201	230.124	2.257	1.622	0.0017	up	ESI+
	Sanguinarine	332.096	314.172	0.314	1.839	0.0174	down	ESI+
	Fenfuram	201.143	238.682	0.368	1.716	0.0296	down	ESI+
	Salvianolic acid D	340.085	24.829	0.335	1.792	0.0091	down	ESI+
FF	3-Hydroxypentanedioic acid	146.965	343.145	2.233	1.529	0.0102	up	ESI−
	(2 S)−2-Amino-3-methylbutanoic acid	116.034	302.698	2.965	1.916	0.0001	up	ESI−
	12-Methyltridecanoic acid	227.021	27.182	2.248	1.753	0.0010	up	ESI−
	12-Keto-leukotriene B4	357.249	552.146	2.369	1.922	0.0003	up	ESI−
	16-Hydroxy hexadecanoic acid	272.097	67.627	0.265	1.732	0.0446	down	ESI−
	(S)−3-Methyl-2-oxopentaoate	129.055	35.895	2.627	1.708	0.0071	up	ESI−
	Hypoxanthine	134.560	179.693	0.049	1.795	0.0385	down	ESI−
	Heptanoic acid	129.496	57.888	2.491	1.772	0.0061	up	ESI−
	9-Decenoic acid	168.978	76.558	0.288	1.749	0.0004	down	ESI−
	Xanthine	150.041	210.954	0.254	1.880	0.0097	down	ESI−
	Threonic acid	134.046	366.756	0.372	1.763	0.0382	down	ESI−
	6-Trans-leukotriene B4	282.049	255.888	2.396	2.083	0.0021	up	ESI−
	4-Methyl-2-oxopentanoate	129.055	57.887	2.364	1.833	0.0019	up	ESI−
	cis-Vaccenic acid	281.099	292.814	0.296	1.998	0.0254	down	ESI−
	Oleoyl glycine	338.042	84.596	2.347	1.652	0.0006	up	ESI−
	2-Hydroxypentanoic acid	117.038	302.727	3.322	1.683	0.0001	up	ESI−
	Cytosine	110.035	255.589	0.408	2.001	0.0128	down	ESI−
	Ginkgoic acid	344.289	31.545	2.181	1.638	0.0347	up	ESI−
	Bilirubin	583.257	58.751	0.161	1.572	0.0360	down	ESI−
	L-Valine	118.151	426.139	0.305	1.823	0.0289	down	ESI−
	Parachlorophenol	126.904	27.189	0.456	1.871	0.0011	down	ESI−
	Hypogeic acid	253.032	77.404	0.201	1.623	0.0078	down	ESI−
	Deoxyinosine	250.949	178.890	0.187	1.618	0.0119	down	ESI−
	Dopamine	152.064	98.584	0.272	1.674	0.0363	down	ESI−
	Alantolactone	230.964	58.128	2.825	1.823	0.0018	up	ESI−
	Thioguanine	167.076	201.053	0.361	1.636	0.0481	up	ESI+
	Thymine	127.050	89.752	0.449	1.596	0.0260	down	ESI+
	Quinoline	130.065	47.975	2.368	1.633	0.0049	down	ESI+
	Melphalan	436.015	26.312	2.027	1.825	0.0002	up	ESI+
	Urocanate	138.938	322.528	0.468	1.737	0.0203	down	ESI+
	3-Methylguanine	166.072	201.246	0.384	1.560	0.0479	down	ESI+
	1-Methyladenosine	282.120	130.661	0.457	1.769	0.0060	down	ESI+
	Lactulose	366.060	446.669	0.182	1.705	0.0273	down	ESI+
	Dihydroneopterin phosphate	335.074	269.055	0.145	1.860	0.0034	down	ESI+
	Oleamide	282.279	75.131	0.167	1.863	0.0182	down	ESI+
	Obtusilactone A	308.295	31.346	0.363	1.585	0.0271	down	ESI+
	Lys Pro Leu	357.249	552.146	0.369	1.655	0.012	down	ESI+
	PE(18:4(6Z,9Z,12Z,15Z)/P-18:1(11Z))	721.557	58.744	0.303	1.679	0.0154	down	ESI+
	LysoPC(14:1(9Z))	465.317	211.144	0.379	1.852	0.0070	down	ESI+
	6-Hydroxy-5-methoxyindole glucuronide	340.103	313.810	2.921	1.590	0.0043	up	ESI+
	PE(18:3(9Z,12Z,15Z)/P-18:1(11Z))	724.530	157.106	0.391	1.726	0.0376	down	ESI+
	3-Indoleacetonitrile	157.076	33.887	2.391	1.530	0.0230	down	ESI+
	1,7-Dimethylguanosine	311.146	193.370	0.266	1.782	0.0213	down	ESI+
	5-Hydroxyisourate	184.037	398.320	0.228	1.737	0.0471	down	ESI+
	PE(P-18:1(11Z)/18:4(6Z,9Z,12Z,15Z))	721.557	169.021	0.356	1.653	0.0310	down	ESI+
	SM(d18:1/22:0)	788.545	223.441	0.117	1.935	0.0176	down	ESI+
	PS(18:0/16:1(9Z))	762.527	224.514	0.082	1.771	0.0452	down	ESI+
	4-Hydroxybenzaldehyde	122.925	322.522	0.484	1.828	0.0142	down	ESI+
	3-Methyl-1,3-dihydroindol-2-one	148.176	25.922	2.091	1.873	0.0010	up	ESI+
	3,5-Digalloylepicatechin	594.139	255.876	0.326	1.889	0.0001	down	ESI+
	PC(P-18:1(9Z)/16:1(9Z))	742.392	232.523	0.256	1.652	0.0406	down	ESI+
Ovarian tissue	Ethyl dodecanoate	227.021	25.595	2.456	2.508	0.0001	up	ESI−
	(2,5-Dioxoimidazolidin-4-yl)urea	156.066	221.761	2.057	2.326	0.0014	up	ESI−
	13-L-Hydroperoxylinoleic acid	311.224	195.159	2.026	2.322	0.0052	up	ESI−
	Benzene-1,2,4-triol	125.000	419.607	9.128	2.796	0.0245	up	ESI−
	12-Methyltridecanoic acid	229.371	27.182	2.248	1.753	0.0010	up	ESI−
	12-Keto-leukotriene B4	335.452	41.107	3.110	1.553	0.0230	up	ESI−
	L-Valine	118.150	414.233	0.305	1.843	0.0073	down	ESI−
	Thr-Gly	176.092	301.131	2.559	2.070	0.0311	up	ESI+
	17beta-Acetamidoandrost-4-en-3-one	329.247	30.475	4.113	2.618	0.0084	up	ESI+
	3-Methyl-1,3-dihydroindol-2-one	148.076	25.035	2.596	2.508	0.0001	up	ESI+
	[10]-Paradol	335.258	35.546	2.475	2.393	0.0132	up	ESI+
	Zalcitabine	212.105	338.783	49.229	2.887	0.0003	up	ESI+
	Amidosulfuron	370.209	450.167	2.734	2.071	0.0166	up	ESI+
	Allantoin	159.120	56.131	0.448	2.177	0.0437	down	ESI+

*FF* follicular fluid, *IO* inactive ovaries, *FC* fold change, *RT* retention time, *FC* fold change, *VIP* variable importance for the projection, *ESI* electrospray ionization

### Pathway analysis

**Fig. 3:**
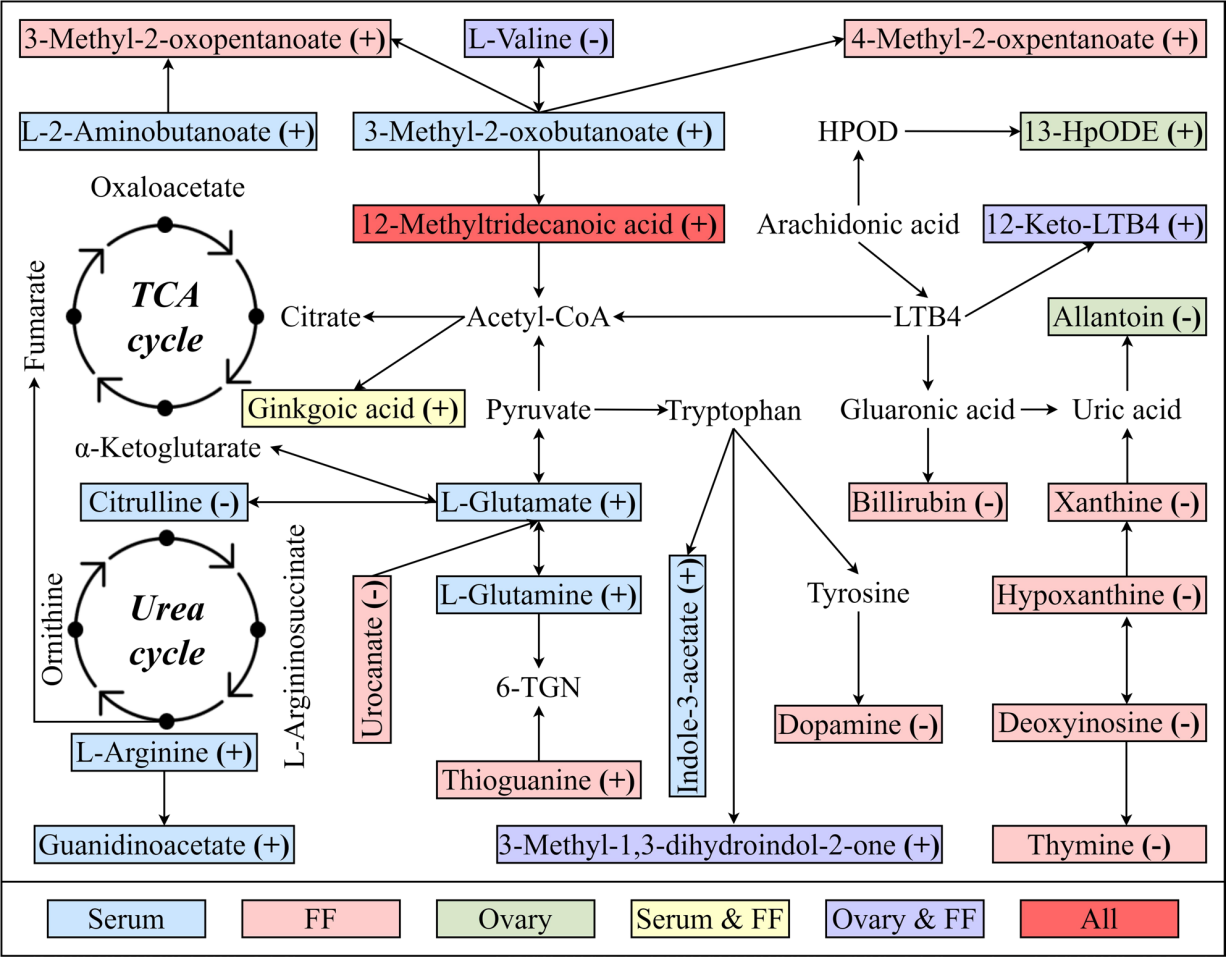
Metabolic network diagram illustrating differential metabolites in dairy cows with inactive ovaries. The symbols (+) and (-) indicate upregulation and downregulation, respectively. Blue represents serum samples, pink represents FF samples, green represents ovarian tissue samples, yellow represents common metabolites in both the serum and FF samples, purple represents common metabolites in both the FF and ovarian tissue samples, and red represents all. FF, follicular fluid; TCA, tricarboxylic acid; 6-TGN, 6-thioguanine nucleotide; LTB4, leukotriene B4; HPOD, hydroperoxy-octadecadienoic acid; 13-HpODE, 13-hydroperoxylinoleic acid

**Fig. 4:**
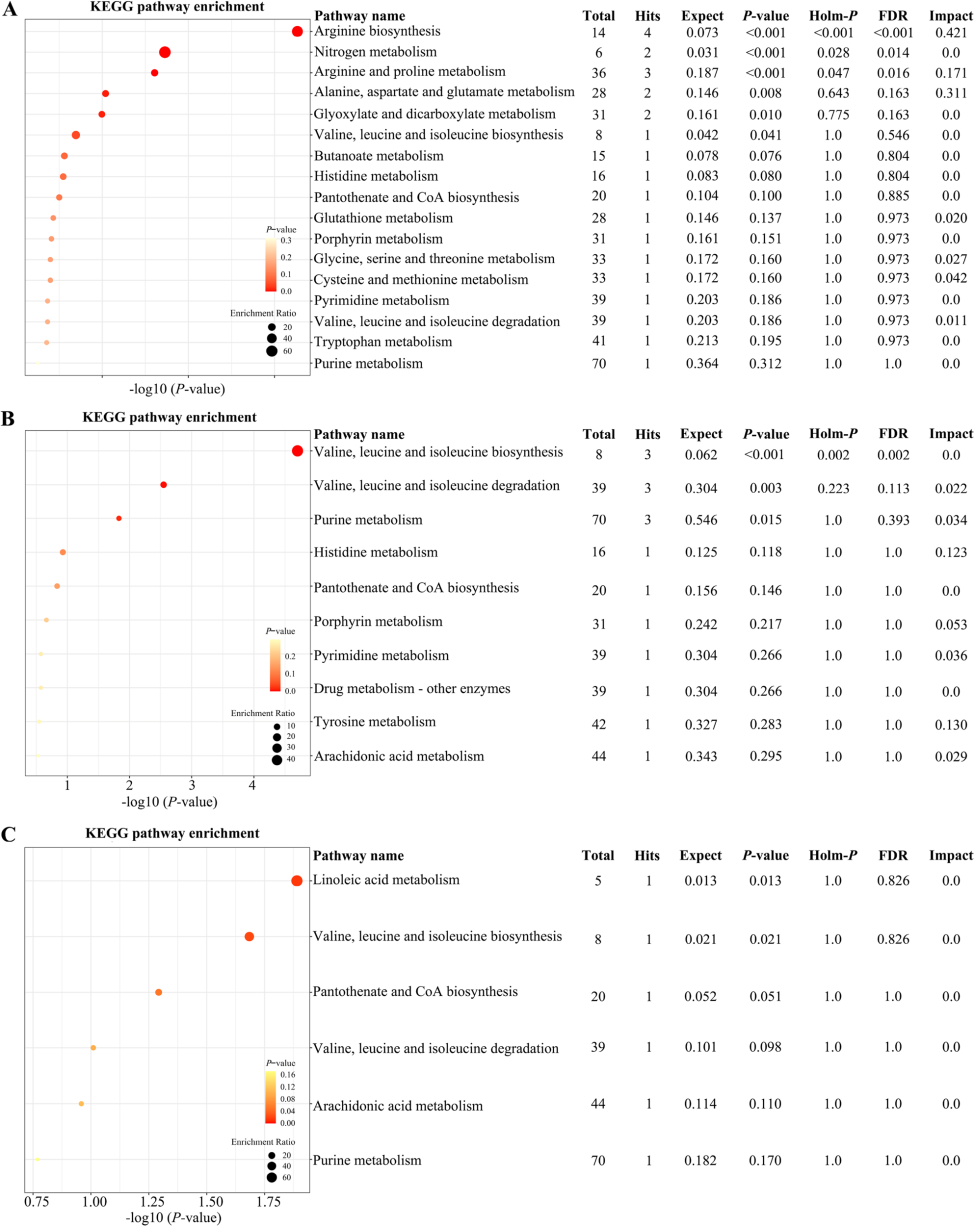
Enrichment bubble map depicting the metabolic pathways associated with differential metabolites identified in serum (**A**), follicular fluid (**B**), and ovarian tissue (**C**) samples. The position and size of the bubble on the abscissa indicate the influence factor of the path in the topological analysis. The larger the bubble, the greater the influence factor. The position and color of each bubble along the x-axis indicate the *p*-value from the enrichment analysis (-log10 *p*-value, or negative logarithm base 10). A smaller *p*-value corresponds to a darker color, indicating more significant enrichment. KEGG, Kyoto Encyclopedia of Genes and Genomes; Total, the total number of compounds in the pathway; Hits, the corresponding number of metabolites in one pathway; Expect, the expected number of matched metabolites observed in the metabolic pathway under random conditions; Holm-*p*, the *p*-value modified utilizing Holm-Bonferroni approach; FDR, the false discovery rate, representing the proportion of results incorrectly identified as significant; Impact, the pathway influence value calculated from pathway topology analysis

Figure 3 illustrates the interaction network of differential metabolites constructed by querying the KEGG pathway database and integrating global metabolic data from published literature. This network highlights the relationships between the differential metabolites identified in the present study. Subsequently, using KEGG pathway analysis, distinct metabolites in the pathways of cows in the IO and HC groups were identified and visualized using a bubble diagram (Fig. 4). Significant changes were identified in six, three, and two metabolic pathways related to IO in the serum, FF, and ovarian tissue samples, respectively. In serum samples, the altered pathways included arginine biosynthesis (*p* < 0.001); nitrogen metabolism (*p* < 0.001); arginine and proline metabolism (*p* < 0.001); alanine, aspartate, and glutamate metabolism (*p* = 0.008); glyoxylate and dicarboxylate metabolism (*p* = 0.010); and valine, leucine, and isoleucine biosynthesis (*p* = 0.041). In FF samples, valine, leucine, and isoleucine biosynthesis (*p* < 0.001); valine, leucine, and isoleucine degradation (*p* = 0.003); and purine metabolism (*p* = 0.015) were found to be altered. In ovarian tissue, the altered pathways included linoleic acid metabolism (*p* = 0.013) and valine, leucine, and isoleucine biosynthesis (*p* = 0.021). Notably, the biosynthesis of valine, leucine, and isoleucine was a metabolic pathway that exhibited significant changes (*p* < 0.05) in serum, FF, and ovarian tissue samples.

## Discussion

Using LC–MS/MS and sophisticated statistical analyses, notable differences in the metabolite profiles were identified among the serum, FF, and ovarian tissue samples, highlighting alterations in various metabolic pathways. Here, we discuss the implications of our findings in different sample types for postpartum IO, and integrate these results to offer a more comprehensive understanding of the metabolic changes associated with postpartum IO (Fig. 3).

Nutritional status is widely acknowledged as a key factor influencing IO because of the strong relationship between postpartum ovarian function and energy balance. Our results showed that cows with IO had a significantly lower BCS and a higher milk yield from their previous lactation than healthy cows. These findings align with earlier studies, indicating that poor BCS and excessive milk yield contribute to the onset of IO [7, 41, 42]. Slow LF growth rate, along with low serum levels of estradiol and progesterone, were also observed in cows with IO (~ 0.57 mm/d), but not in healthy cows (~ 1.26 mm/d), which aligns with the known characteristics of follicular dynamics in IO [27, 43]. The serum level of NEFA indicates the extent of fat mobilization [44]. An elevation in circulating NEFA is frequently a reliable marker of NEB in dairy cows [45]. The IGF-1 is essential for gonadotropin-induced folliculogenesis, ovarian steroidogenesis, and the functioning of the corpus luteum. Additionally, it regulates the functions of both the pituitary and hypothalamus [46]. Serum AST levels are routinely used in the test of liver function to assess liver damage. Cows with IO generally exhibit poor liver function [47]. Zulu et al. (2002) [48] reported that cows with IO had elevated serum levels of NEFA and AST, along with decreased levels of IGF-1 compared to normal cows. These findings align with our results in cows with IO.

The specific roles of calcium and phosphorus in IO in cows have not yet been reported. In the current study, cows with IO had significantly lower serum calcium and phosphorus levels than healthy cows; however, these levels were still within the normal range: calcium (2.07–2.59 mmol/L) and phosphorus (1.36–2.49 mmol/L) [49]. Recent study on cows with IO revealed presence of non-significant variations in calcium and phosphorus levels between cows in estrus compared to cows with IO [5]. However, another study on buffaloes showed that mean serum phosphorus was found decreased in buffaloes suffered from postpartum IO, while its levels showed increasing trend when the buffaloes came into oestrus and became pregnant after controlled internal drug release treatment [50]. Thus, it is reasonable to speculate that insufficient levels of calcium and phosphorus may play a role in the onset of IO. Collectively, IO in postpartum cows may be attributed to multiple factors, such as prolonged and more severe NEB; inadequate BCS and compromised liver function; excessive milk yield and fat mobilization; and low circulating IGF-1, calcium, and phosphorus concentrations during early lactation. Nevertheless, the synergistic mechanisms underlying several factors involved in postpartum IO require further investigation.

Multiple metabolomics studies have demonstrated that postpartum IO is associated with alterations in amino acid, lipid, and carbohydrate metabolism in the serum or plasma of dairy cows [9, 10, 14, 27]. In the present study, six pathways related to amino acid, energy, and carbohydrate metabolism were found to be significantly altered in the serum of cows with IO. Eight metabolites involved in these pathways were identified in the IO group. A previous study showed that circulating L-glutamate and L-glutamine levels are higher in cows with IO than in estrous cows [27], which is consistent with our results. L-glutamine is the preferred precursor of the major excitatory neurotransmitter, L-glutamate [51]. Both can be interconverted enzymatically and play crucial roles in amino acid, energy, and carbohydrate metabolism [52]. A recent study demonstrated that L-glutamate injection promoted follicular development in goats with low BCS [53]. On one hand, L-glutamate can be transformed into α-ketoglutarate, allowing it to enter the tricarboxylic acid cycle (TCA) to provide energy for follicular development [27]. On the other hand, L-glutamine is also an important precursor for the synthesis of GSH, a key antioxidant. Here, the elevated levels of serum L-glutamine and L-glutamate suggest that both processes may be inhibited in cows with IO, leading to an insufficient energy supply and a lack of antioxidants. Our findings also support the conclusions of previous studies that cows with IO often experience NEB and oxidative stress [7, 47].

Citrulline serves as a precursor in the synthesis of L-arginine in the urea cycle, which produces nitric oxide (NO). In cows with IO, high L-arginine and low citrulline levels in the serum suggest urea cycle dysregulation, which reduces NO synthesis. A reduction in NO levels is not conducive to ovarian blood flow and is linked to steroidogenesis, oxidative stress, and the apoptosis of granulosa cells, which can ultimately damage ovarian activity and function [54, 55]. Guanidinoacetate, an intermediate of arginine metabolism, is a direct precursor of creatinine. Elevated serum guanidinoacetate levels in cows with IO may enhance energy storage and decrease fat mobilization, thereby supporting ovarian activity under NEB and low BCS conditions. Recent studies have established links between 3-methyl-2-oxobutanoate (an L-valine metabolite) and both liver and myocardial injury [56, 57]. Elevated levels of serum 3-methyl-2-oxobutanoic acid in cows with IO may be related to L-valine degradation in the FF and ovarian tissue, and poor liver function. L-alpha-aminobutyric acid, commonly referred to as L-homoalanine, is a non-proteinogenic α-amino acid that has recently been found to have considerable potential for inhibiting inflammation [58, 59]. Cows with IO usually exhibit an increased inflammatory response [47]. Elevated serum l-alpha-aminobutyric acid levels in cows with IO may be a compensatory response to inflammation. Dietary tryptophan is metabolized into indole-3-acetate by the gut microbiota [60]. Previous studies have revealed alterations in the levels of various bacterial species from the genera *Lactobacillus*, *Bacteroides*, and *Streptococcus* when exposed to stress conditions [61–65]. Thus, elevated serum indole-3-acetate levels in cows with IO may be a response to stress-induced intestinal floral disorders. This is a notable finding given established the connection between gut microbiota and the development of antral follicles in Holstein cows [66].

Multiple metabolomic investigations have demonstrated that postpartum IO is linked to changes in the metabolism of amino acid, lipid, and carbohydrate in the FF of dairy cows [27, 67]. In the present study, three pathways associated with amino acid and nucleotide metabolism were significantly altered in the FF of cows with IO. Twelve primary metabolites involved in these pathways were identified in the IO group. Branched-chain amino acids (BCAAs) are essential for in protein synthesis, energy metabolism, and hormone regulation [68]. L-Valine levels in FF decreased, whereas the degradation products (4-methyl-2-oxopentanoate and [S]−3-methyl-2-oxopentanoate) of two other BCAAs were elevated in cows with IO in this study. This aligns with earlier research showing that plasma BCAA levels are significantly decreased in anestrus cows with NEB [14]. Deoxyinosine, hypoxanthine, and xanthine are integral to purine metabolism, whereas thymine is associated with pyrimidine metabolism. All these compounds are involved in nucleotide metabolism. Reduced levels of these metabolites in the FF of cows with IO reflect a disorder of nucleotide metabolism, which has previously been linked to adverse effects on follicular development and oocyte quality [69]. Thioguanine, an analog of the physiological purines guanine and hypoxanthine, functions as a purine anti-metabolite. In our study, thioguanine levels in the FF of cows with IO exhibited a trend opposite to that of hypoxanthine levels, supporting a competitive relationship between the two [70]. Bilirubin, a product of heme catabolism, possesses strong antioxidant and anti-inflammatory effects. In this study, a notable decrease was observed in the bilirubin levels within the FF of cows exhibiting IO. This finding supports the findings of a previous study showing that cows with IO have weakened anti-inflammatory and antioxidant capacities [27]. Urocanate is a histidine degradation product with local and systemic anti-inflammatory and immunosuppressive properties [71]. Reduced urocanate levels in the FF of cows with IO may explain their decreased immunosuppressive abilities. 12-Keto-LTB4, a metabolite of arachidonic acid, is converted from the intermediate LTB4. The production of 12-keto-LTB4 signals the inactivation of LTB4, which is an inflammatory mediator [72]. Thus, the elevated 12-keto-LTB4 levels in the FF of cows with IO reflect the metabolic regulatory capacity of cells in response to inflammation. Dopamine is an important neurotransmitter that regulates the secretion of reproductive hormones, such as prolactin, FSH, and LH [73, 74]. When dopamine levels decrease, prolactin secretion increases, further suppressing FSH secretion, which contributes to follicular dysplasia and ultimately leads to IO [75, 76]. Thus, low dopamine levels may be a valuable biomarker in the FF of cows with IO.

The metabolomic characteristics underlying postpartum IO in the ovarian tissues of cows have not been characterized because of challenges associated with sample acquisition. To our knowledge, this study represents the first investigation into the metabolic profile of ovarian tissue in cows with IO. We found that alterations in lipid, amino acid, nucleotide, cofactor, and vitamin metabolism within the ovarian tissue were associated with IO. Six relevant metabolic pathways were identified. Linoleic acid metabolism (lipid-related) and the biosynthesis of valine, leucine, and isoleucine (amino acid-related) exhibited significant changes in the ovarian tissue of cows with IO. Four major differential metabolites involved in these pathways were identified in the IO group. 13-Hydroperoxyoctadecadienoic acid (13-HpODE), a lipid peroxidation product of linoleic acid, is a potential marker of oxidative stress. Hennet et al. (2013) [77] found that the concentration of FF 13-HpODE was notably lower in the first subordinate follicles compared to the second subordinate follicles within each pair of ovaries. Elevated levels of 13-HpODE indicated significant oxidative stress and lipid damage in the ovarian tissue of cows with IO, inhibiting oocyte maturation and subsequent development [78]. L-Valine can be metabolized to succinyl-CoA, an intermediate of the TCA cycle, which is crucial for adenosine triphosphate (ATP) production. Adequate ATP levels are essential for maintaining the energy balance in ovarian cells and supporting processes, such as follicular development and oocyte maturation [79]. Conversely, L-valine deficiency observed in both the FF and ovarian tissues of cows with IO can reduce ATP production, thereby impairing these vital reproductive processes. 12-Keto-LTB4 levels in the ovarian tissue of cows with IO showed a trend similar to that observed in the FF of these cows. As previously discussed, elevated levels of 12-keto-LTB4 in the ovarian tissue of cows with IO indicate that these cells possess a metabolic regulatory capacity in response to inflammation. Allantoin, a product of purine metabolism, has anti-inflammatory and antioxidant properties that promote cell growth. It has recently been reported that allantoin can alleviate cyclophosphamide-induced premature ovarian failure in female rats, associated with a reduction in anestrum; increased estradiol levels; decreased FSH and LH levels; ameliorated apoptosis; and decreased mitochondrial membrane potential, reactive oxygen species levels, and mitophagy in ovarian granulosa cells [80]. Reduced allantoin levels in the ovarian tissues of cows with IO may be a response to increased oxidative stress.

A novel finding of our study was that 12-methyltridecanoic acid was significantly upregulated in the serum, FF, and ovarian tissues of cows with IO. 12-Methyltridecanoic acid is a branched-chain saturated fatty acid and an important component of cow milk fat. While these findings indicate that 12-methyltridecanoic acid emerges as a candidate diagnostic biomarker for IO, its clinical utility mandates rigorous validation through multicenter prospective studies with large heterogeneous cohorts. Another novel finding of our study is that valine, leucine, and isoleucine biosynthesis exhibited significant alterations in the serum, FF, and ovarian tissues of cows with IO. Given the positive relationship between BCAAs and follicular maturation, the selective targeting of the BCAA biosynthetic pathway may be an effective method for treating IO [81]. One puzzling observation is that ginkgoic acid was significantly upregulated in the serum and FF of cows with IO, despite the fact that ginkgo extract has been sanctioned for use as a feed additive [82]. 3-Methyl-1,3-dihydroindol-2-one (also known as 3-methyloxindole) is a metabolite of L-tryptophan that is formed in the rumen of cattle [83]. Elevated levels of 3-methyloxindole in both the FF and ovarian tissues of cows with IO may suggest changes in gut microbes; however, the levels of 3-methyloxindole were not notably elevated in the circulation of the cows with IO. Regrettably, the mechanism underlying this phenomenon remains unclear. Collectively, these metabolic changes underscore the importance of maintaining an energy and nutritional balance for optimal ovarian function. The comprehensive response to metabolic, oxidative, and inflammatory stresses, as evidenced by the altered levels of these metabolites, highlights the complexity of postpartum IO and the need for targeted metabolic support to improve reproductive outcomes in cows under these conditions.

While this study provides a comprehensive metabolomic analysis of postpartum IO in dairy cows, several limitations must be acknowledged. The small sample size (*n* = 6/group) was determined post-hoc based on resource availability rather than a priori power analysis. While the findings provide biologically relevant insights, future studies with larger cohorts are warranted to strengthen statistical robustness. The IO group was selected based on ultrasonography and progesterone levels but may include heterogeneous underlying conditions (especially NEB). Future studies should stratify IO cases by etiology to clarify whether metabolic changes reflect IO itself or upstream conditions. The control group consisted solely of luteal-phase cows under progesterone influence, which may confound comparisons with estradiol-dominant states. Additionally, the Presynch-Ovsynch protocol, while standardizing follicular waves, could amplify differences between groups. Despite these constraints, our multi-tissue approach offers novel insights into IO-associated metabolic dysregulation. Further research should include follicular-phase controls and mechanistic studies to distinguish hormonal from intrinsic metabolic effects. These refinements will enhance the clinical applicability of our findings.

## Conclusions

Untargeted metabolomics facilitates the detection of systemic metabolic changes in dairy cows with postpartum IO. In this study, LC–MS/MS analysis of serum, FF, and ovarian tissues identified 40, 51, and 14 differential metabolites, respectively, and highlighted alterations in nine key metabolic pathways. These findings reflect a complex response to metabolic, oxidative, and inflammatory stresses, providing novel insights into IO-associated metabolic dysregulation and highlighting potential biomarkers for early diagnosis and therapeutic targets. Future research should focus on validating biomarkers and elucidating underlying mechanisms to improve reproductive outcomes.

## Data Availability

The data that support the findings of this study are available from the corresponding authors upon reasonable request.

## Abbreviations

IO: Inactive ovaries
FF: Follicular fluid
HC: Healthy control
LF: Largest follicle
DIM: Days in milk
BCS: Body condition score
NEB: Negative energy balance
LC-MS/MS: Liquid chromatography-tandem mass spectrometry
TMR: Total mixed ration
PGF_2a_: Prostaglandin F2 alpha
GnRH: Gonadotropin-releasing hormone
NEFA: Non-esterified fatty acids
AST: Aspartate aminotransferase
ALT: Alanine aminotransferase
TP: Total protein
IGF-1: Insulin-like growth factor 1
PCA: Principal component analysis
OPLS-DA: Orthogonal partial least squares discriminant analysis
VIP: Variable importance for the projection
FC: Fold change
FSH: Follicle-stimulating hormone
LH: Luteinizing hormone
TCA: Tricarboxylic acid cycle
NO: Nitric oxide
BCAAs: Branched-chain amino acids
13-HpODE: 13-Hydroperoxyoctadecadienoic acid
ATP: Adenosine triphosphate
